# Exploring superior capsular reconstruction and tendon transfers for massive irreparable posterosuperior rotator cuff tears

**DOI:** 10.1530/EOR-2024-0139

**Published:** 2025-09-04

**Authors:** Majd Mzeihem, Mohamad Nassereddine, Anas El Zouhbi, Farid Amirouche, Bassem Elhassan

**Affiliations:** ^1^ Department of Orthopaedic Surgery, University of Illinois at Chicago, Chicago, Illinois, USA; ^2^ Department of Orthopedic Surgery, American University of Beirut Medical Center, Beirut, Lebanon; ^3^ Department of Orthopaedic Surgery, Northshore University Health System, An Affiliate of The University of Chicago Pritzker School of Medicine, Skokie, Illinois, USA; ^4^ Department of Orthopaedic Surgery, Massachusetts General Hospital, Boston, Massachusetts, USA

**Keywords:** rotator cuff, rotator cuff tears, superior capsular reconstruction, tendon transfer, shoulder pathology, shoulder surgery

## Abstract

- Rotator cuff tears are prevalent, affecting 20% of the general population, with massive tears accounting for 40% of these cases. Massive tears, those larger than 5 cm or involving several tendons, pose substantial clinical problems, including poorer surgical outcomes and increased recurrence rates.
- Multiple classification systems offer varied definitions, complicating treatment strategies. The irreparability of these tears, exacerbated by conditions such as tendon atrophy and advanced imaging abnormalities, can further complicate management.
- Surgical options include superior capsular reconstruction (SCR) and tendon transfers. SCR, which involves attaching a graft to the superior glenoid and greater tuberosity, has shown promise in individuals with intact subscapularis tendons and minimal arthritis. Graft alternatives include fascia lata (FL) autografts, human dermal allografts, and long head of the biceps tendon (LHBT) autografts. Each graft type has distinct advantages and disadvantages, with FL autografts providing greater results despite donor site morbidity.
- Tendon transfers, such as latissimus dorsi and lower trapezius transfers, offer alternative treatments, especially for younger, more active individuals. This review thoroughly reviews different therapeutic options, emphasizing the most recent evidence and clinical outcomes to help guide the best management of massive posterosuperior irreparable rotator cuff injuries.

## Introduction

Rotator cuff tear (RCT) is one of the most common musculoskeletal injuries seen by orthopedic surgeons, with a prevalence of 20% of the general population (1). Of all RCTs, 40% are classified as massive tears (2). Massive tears have been defined in the literature as those with either a maximum diameter of >5 cm or tears that involve two or more tendons (3, 4). The tear size is essential when considering clinical outcomes after surgical intervention. For instance, the repair of massive tears is more prone to poorer outcomes, structural failure, delayed healing, the potential of irreparability, and a higher rate of re-tear after surgical repair, ranging from 18 to 94% in recent studies (5, 6).

To achieve optimal outcomes, physicians must be well-versed in treatment options, indications, biomechanics, and outcomes. Massive RCTs are defined differently by physicians, and various classification systems have been proposed to guide treatment choices. Cofield and DeOria describe them as tears with a ≥5 cm diameter in the anterior-posterior or medial–lateral planes (7). Gerber defines them as complete tears of ≥2 tendons from their tuberosities (8). Davidson and Burkhart link RCT patterns to prognosis, defining massive tears as contracted >2 × 2 cm in sagittal and coronal dimensions (9). Schumaier *et al.* generated a Delphi consensus in 2019, defining massive tears as retractions to the glenoid rim in axial or coronal dimensions, or exposure of two-thirds of the greater tuberosity in the sagittal plane (6).

Furthermore, defining an irreparable RCT is crucial, given the multiple factors that could affect the tendon’s irreparability status. Irreparability is defined as the inability of the rotator cuff tendon to attach to the footprint during attempted repair and failure of mobilization due to it being so contracted or atrophied (10). The concept of irreparability depends on preoperative advanced imaging and clinical characteristics that can theoretically predict repair success. Findings suggestive of irreparability include a positive tangent sign on magnetic resonance imaging (MRI), Goutailler grade 3–4 fatty infiltration, superior migration of the humeral head with an acromiohumeral interval less than 6 mm, U-shaped tear, and chronic pseudoparalysis (11, 12, 13, 14).

**Figure 1:**
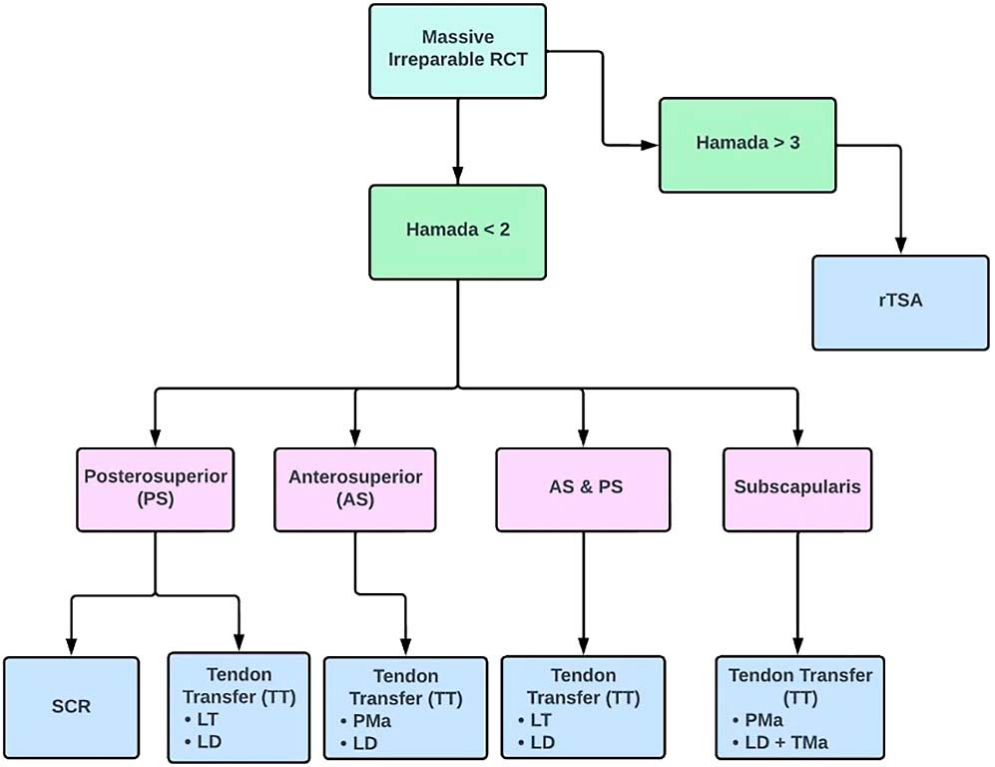
Flow chart for the management of irreparable MRCT. rTSA, reverse total shoulder arthroplasty; LT, lower trapezius; LD, latissimus dorsi; PMa, pectoralis major; TMa, teres major.

The management of massive irreparable RCT remains a challenge, with diverse treatment options lacking a universally superior treatment (10). Conservative interventions such as physical therapy, injections, and NSAIDs often fall short of delivering adequate pain relief and restoring functional capacity. Figure 1 depicts a flowchart based on the published literature by leading scholars in shoulder surgery, suggesting an algorithm for treating irreparable RCTs based on the Hamada classification (15).

This review aims to provide a comprehensive update on the various treatments for irreparable massive rotator cuff tears (MRCT). We will describe the latest evidence on superior capsular reconstruction (SCR), considering both fascia-lata and non-fascia-lata grafts, and explore tendon transfers used to repair posterosuperior MRCT.

## Superior capsular reconstruction

### Definition

**Figure 2:**
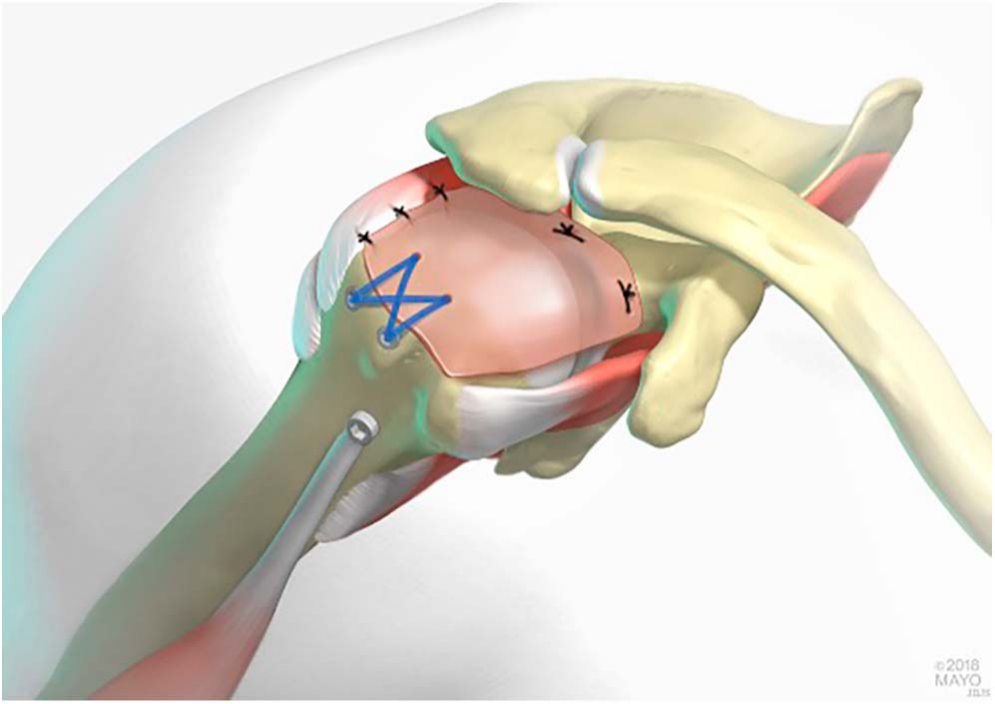
Illustration of superior capsular reconstruction (Reproduced with permission from Burnier *et al*. (69); used with permission of Mayo Foundation for Medical Education and Research. All rights reserved).

SCR is a surgical approach for managing MRCT, first described by Mihata *et al.* (16). It consists initially of anchoring a fascia lata autograft at the surface of the superior glenoid medially and the greater tuberosity cuff footprint laterally. The graft is then attached to the infraspinatus and supraspinatus or the subscapularis tendon using the side-to-side suturing technique for better fixation, as shown in Fig. 2 (16).

### Indications

Indications for SCR include cases with an MRCT, where the subscapularis tendon is either intact or repairable. Criteria for consideration include patients who have an irreparable tear without a severe bone deformity (i.e. Hamada V patients), severe superior migration of the humeral head (which does not move with arm traction), cervical nerve palsy, axillary nerve palsy, deltoid muscle dysfunction, or infection (16, 17). Intolerable pain persisting for at least 6 weeks despite conservative treatment, along with minimal or absent signs of arthritis on both anteroposterior (AP) and axillary imaging (18). Frank *et al.* highlight the necessity for unacceptable dysfunction and non-compromised deltoid muscle function as part of the indications (19). Pasqualini *et al.* have published a treatment algorithm for irreparable RCT, suggesting SCR as a viable option in cases with significant fatty infiltration without significant and advanced degenerative changes (20).

### Surgical technique

The technique starts by measuring the defect’s size in the sagittal–coronal plane intraoperatively with the arm 30° abducted and 10–15° externally rotated. Before inserting the anchor, the superior glenoid surface is prepared with a mechanical reamer to increase its bleeding surface, promoting graft healing. Graft fixation in the glenoid typically uses two anchors (anterior at 2 o’clock and posterior at 10 o’clock) via the Neviaser portal (21). To our knowledge, the impact of the number of anchors on clinical outcomes does not play a significant role. Some biomechanical cadaver studies show that 3-point fixation improves glenohumeral stability, reduces subacromial pressure, and enhances superior translation compared to 2-point fixation. In addition, three anchors outperform in peak load, energy, stiffness, and yield load (22, 23, 24). There is no consensus on anchor placement; Pennington *et al.* suggest placing anchors at the superior glenoid rim and joint margin for better fixation (25), while other studies advocate for medial placement along the glenoid neck (26, 27, 28).

The common method for graft fixation to the greater tuberosity in SCR involves double-row sutures, using two medial and two lateral row anchors for transosseous fixation. One study reported a 36.1% graft failure rate with single-row SCR graft fixation, as determined by MRI (29). No clinical studies have directly compared both fixation techniques, but this study suggests that double-row transosseous fixation is the preferred approach for graft fixation to the greater tuberosity.

Opinions on graft management during arthroscopic SCR vary. Some studies recommend passing sutures outside the shoulder before graft implementation to avoid entanglement (28, 30, 31, 32, 33). However, this technique complicates graft tensioning, requiring precise measurements between suture anchors, as tension adjustments are not possible once the graft is introduced (21). Alternatively, others suggest passing sutures after graft placement in the shoulder to allow better tensioning (25, 34, 35, 36, 37).

## Graft types

### Fascia lata (FL) autograft

Various grafts can be used to reconstruct the superior glenohumeral joint’s superior capsule. FL autografts, initially described by Mihata *et al.* involve harvesting a graft 2–3 times the defect’s size, with a 6–8 mm thickness after multiple folds. Their study demonstrated that using an 8 mm FL autograft significantly reduces peak subacromial pressure by 246 and 158%, with decreased superior translation by 135 and 130% at 0 and 30 degrees of abduction, respectively, after SCR. Some studies suggest enhanced glenohumeral joint stability with thicker grafts (8 vs 4 mm) at 10–30 degrees of abduction (38). However, concerns about donor site morbidity often deter physicians from choosing FL grafts. A minimally invasive harvesting technique has been proposed, involving two transverse 2 cm thigh incisions (39, 40). Clinical studies in the literature indicate minimal to no impact on thigh morbidity after the use of such a technique. For example, in 39 patients undergoing SCR with minimally invasive FL graft harvesting, the Western Ontario and McMaster Universities Osteoarthritis Index (WOMAC) score difference was 0.8% compared to the contralateral thigh after 47 months of follow-up, with no significant differences in WOMAC or VAC scores (*P* = 0.684 and *P* = 0.148, respectively) (40).

In addition, there is an ongoing debate concerning graft thickness in the minimally invasive technique, with some arguing that it results in a thinner graft (5 mm) after folding, and attempts to obtain a thicker graft may lead to a relatively large donor defect (21). Despite this, both harvesting techniques have demonstrated similar positive clinical outcomes (16, 39, 41). A cadaveric study conducted in 2021 compared openly harvested proximal thigh and minimally invasively harvested mid-thigh FL autografts, revealing biomechanical equivalence in terms of stiffness and Young’s modulus (*P* = 0.415 and *P* = 0.185, respectively). However, there was a significant difference in graft thickness between the open proximal FL graft and the minimally invasively harvested mid-thigh graft (7.17 ± 1.97 mm vs 5.54 ± 1.37 mm). Consequently, the minimally invasive mid-thigh harvesting technique may hold an advantage over the open proximal thigh technique due to its reproducibility, biomechanical equivalence of the grafts, low donor site morbidity, and preservation of the postural function of the tensor FL proximally and the iliotibial band distally and posteriorly, an important anterolateral knee stabilizer (42, 43). Notably, the minimally invasive technique is not considered more technically demanding than the open approach.

According to the current literature, using FL graft in SCR presents a re-tear rate ranging from 4.5 to 29%. The introduction of propylene mesh, which is commonly used in hernia repair and shoulder joints after endoprosthetic replacement for treating malignant tumors (44, 45, 46), with SCR aims to enhance tensile strength. This addition was initially designed to reduce graft failure rates and improve functional outcomes in patients with MRCT. A cohort study by Kholinne *et al.* demonstrated significant improvements in the ASES score (29.1 ± 15.8 vs 18.1 ± 15.9), shoulder active range of motion (forward flexion: 40 ± 26 vs 28 ± 23, and external rotation: 11 ± 5 vs 6 ± 3) and graft healing rate (83.3 vs 58.8%; *P* = 0.04) when comparing autograft FL SCR augmented with a propylene mesh versus non-augmented with a propylene mesh (47). These findings suggest that mesh augmentation may be a viable addition to conventional SCR as it reduces graft failure and improves clinical outcomes.

### Non-fascia lata (Non-FL) graft

• Dermal allograft:

The development of SCR using human dermal allograft (HDA) aims to avoid the donor site morbidity associated with FL autograft harvesting (31, 48). This technique offers additional advantages: simplified graft preparation, consistent thickness and construct, minimal immunological risk, and effective biological integration with well-maintained integrity. Snyder *et al.*’s histologic study demonstrated that acellular human dermal grafts exhibited excellent incorporation with surrounding cuff tissues, fostering neovascularization and collagen fiber reorganization with minimal inflammatory response (49). This suggests that dermal allografts serve as a scaffold for remodeling the shoulder’s superior capsule, ensuring integrity by incorporating surrounding host tissue with minimal immunological risk.

Several studies have explored the biomechanical properties of using HDA in SCR. One such study compared acellular dermal allografts (aDA) with two different thicknesses in SCR, showing that the 6 mm thick graft provided significantly better restoration of normal glenohumeral joint position and forces (glenohumeral superior translation, subacromial peak contact pressure, and cumulative deltoid force) compared to the 3 mm thick for treating irreparable RCT (50). Cline *et al.*’s cadaveric study comparing FL (7.3 mm thick), double-layered aDA (6.4 mm thick), and single-layered aDA (3.5 mm thick) for SCR demonstrated that all were capable of restoring superior glenohumeral translation, subacromial contact pressure, and a standard range of glenohumeral abduction angle. However, the first two were significantly more effective than the single-layer aDA, particularly in restoring superior translation at 30 and 60 degrees of abduction, and subacromial contact pressure at 0 degrees. In addition, the single layer showed a decrease in graft thickness during testing compared to the others. This study also validated Mihata *et al.*’s graft thickness suggestions by confirming that increasing graft thickness above the 4 mm mark improves SCR’s effectiveness and clinical outcomes (51).

In addition to histologic and cadaveric success, arthroscopic SCR using HDA has demonstrated short- to midterm clinical and functional improvements (18, 52, 53). A multicenter cohort study reported successful outcomes in 70% of patients after arthroscopic SCR using HDA. Among the 59 patients (mean age: 62 years, follow-up: 17.7 months), forward flexion improved from 130° preoperatively to 158° postoperatively, while external rotation improved from 36° to 45° (*P* <0.001). The mean American Shoulder and Elbow Surgeons (ASES) score (43.6–77.5) and the subjective shoulder value (35.0–76.3) improved postoperatively, and the visual analog scale (VAS) decreased from 5.8 to 1.7 (*P* <0.001) (28).

The primary drawbacks of using HDA compared to FL are the increased financial costs associated with the graft itself, the higher risk of infection, and the risk of graft rejection (54, 55).

Multiple studies have compared FL to HDA in SCR. A meta-analysis revealed an increase in ASES score following SCR with a +36-points improvement for HDA and a +64-points improvement for FL autograft. Graft tear and revision surgery rates were 7.9 and 2.6% with HDA, respectively, and 5.6 and 5.7% with FL autograft, respectively (10). Another review reported a higher graft tear rate range with HDA in SCR compared to FL autograft (20–75% vs 5–32%, respectively). In a subgroup analysis, SCR using FL achieved superior humeral translation restoration significantly better than HDA (*P* = 0.02). Both grafts restored subacromial contact pressure, with no significant difference in subacromial peak contact pressure between them (56).

• Long Head of the Biceps Tendon (LHBT) autograft:

Alternative grafting options for SCR include the Long Head of the Biceps Tendon (LHBT) autograft (57, 58, 59). The arthroscopic biceps Chillemi’s technique, described in 2018, shares similarities with the ‘Chinese Way’, differing primarily in the number of required portals and the lateral fixation of the tendon using either two anchors or two transosseous tunnels. The proximal end of the distal biceps tendon is either tenodized or left floating freely, which could result in Popeye’s sign. LHBT offers advantages such as minimal donor site morbidity, reduced cost, graft vitality by retaining its proximal origin and elimination of the need for fixation, thereby simplifying surgery and reducing operative time.

The mean diameter of the LHBT is 6.6 mm (60). Concerns about its width compared to FL or HDA question its ability to cover enough surface, potentially allowing superior humeral migration. However, recent biomechanical studies suggest LHBT is equivalent to FL in preventing superior humeral migration, re-centering the humeral head on the glenoid, and restoring subacromial contact pressure (61, 62, 63, 64). With a mechanical strength of 32.5 ± 5.3 MPa, it is considered sufficient for maintaining integrity (60).

Systematic reviews highlight that the mean VAS post-LHBT SCR exceeds the minimal clinically significant difference (MCID) for adequate pain relief (46). Functional outcomes, including Constant, ASES, and Simple Shoulder Test scores, as well as range of motion, particularly forward elevation and external rotation, significantly improve post-surgery (65, 66, 67). Kocaoglu *et al.* compared SCR for partial RCT using LHBT (14 patients, mean age 64.6 ± 8.4) or FL (12 patients, mean age 62.5 ± 6.5) autografts. Both groups showed significant improvement at a minimum 2-year follow-up, with no significant differences in clinical scores, range of motion, acromiohumeral distance, or re-tear rates. However, the lack of randomization and small sample size require cautious interpretation of these results.

Disadvantages of this technique include potential inflammation of the LHBT due to tension on the proximal insertion caused by SCR. This stress might lead to continuous residual postoperative pain, although the literature results suggest it is not significant. In addition, LHBT cannot be used in severe tendinitis, partial tear, SLAP lesion >II, or the tendon’s scarce anatomical variations or absence.

A systematic review comparing three graft types revealed that FL provided significantly better superior translation restraint than HDA and LHBT (*P*-value = 0.02) (56). FL also had a significantly smaller change in graft material length compared to HDA, with no significant difference in graft thickness. However, the reduction in subacromial contact peak pressure was consistent across graft types used for SCR. Despite donor site morbidity and longer recovery times, FL remains the preferred option for SCR, particularly with the minimally invasive technique that reduces donor site morbidity and the use of propylene mesh, which decreases graft failure rates.

## Tendon transfer

More than 30 years ago, Gerber introduced tendon transfers as a joint-preserving treatment option for irreparable RCT for active patients (68). Any muscle-tendon units around the glenohumeral joint may be considered for tendon transfer to the greater or lesser tuberosity to treat irreparable RCT. These muscle-tendon units provide a local vascularized autograft with a tenodesis effect and powered tendon fibers. However, the strength of the transferred tendon unit is, at best, one lower level compared to the native muscle unit (69). Many tendons used for transfer have been described, including latissimus dorsi (LD), lower trapezius (LT), pectoralis major (PM), and teres major (TM), with tendon selection based on the cuff tear location or functional status. Most can be performed arthroscopically or with arthroscopic assistance (70). This review will only focus on posterosuperior massive irreparable RCT and possible tendon transfer options. A summary of those procedures is highlighted in Table 1.

**Table 1:** Summary of the different tendon transfers used for posterosuperior (supraspinatus or infraspinatus) irreparable MRCT (94, 95).

Tendon transfer	Insertion	Mechanism of action	Benefits	Drawbacks
Latissimus dorsi	The lateral aspect of the greater tuberosity	Restores forward elevation and external rotation	Well-established procedure in the literature	Must have intact subscapularis
				Biofeedback training necessary
Lower trapezius	Greater tuberosity	Restores forward elevation and external rotation	The lower trapezius tendon has the same transfer vector as the infraspinatus	It is more costly if achilles allograft is needed
			No need for biofeedback training	

### Indications for tendon transfers

LD transfer is indicated for massive or irreparable posterosuperior RCT, and conservative treatments have failed. It is suitable for patients without significant glenohumeral arthritis or subscapularis deficiency, and requires a functional deltoid muscle and the absence of axillary nerve palsy (71, 72). LT tendon transfer is indicated for massive, irreparable posterosuperior RCTs with significant external rotation weakness or lag signs, particularly in younger, active patients without glenohumeral arthritis. It is preferred when there is an intact or reparable subscapularis tendon and a functional deltoid muscle, and it is contraindicated in patients with cuff tear arthropathy, combined loss of elevation and external rotation, irreparable subscapularis tear, teres minor involvement, or those unable to comply with rigid rehabilitation guidelines (73).

### Latissimus dorsi (LD) tendon transfer

**Figure 3:**
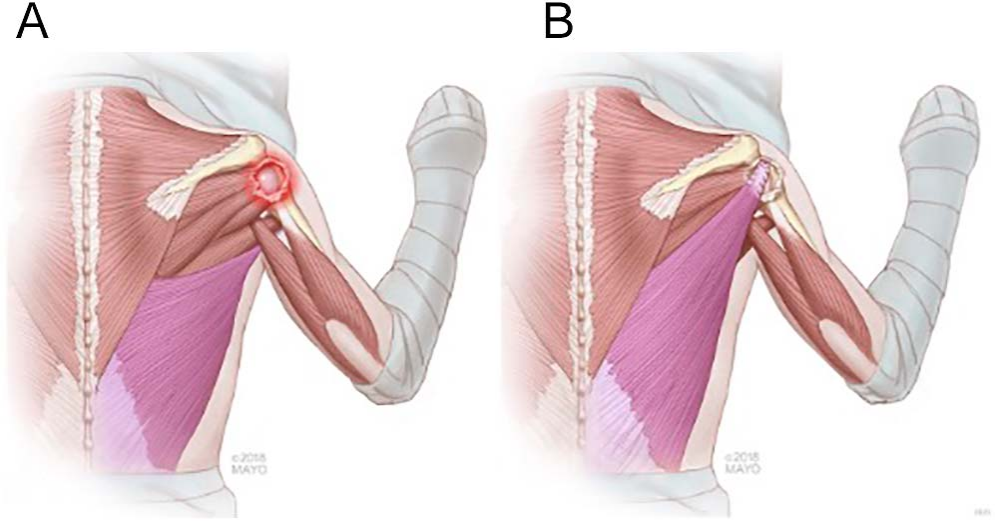
Illustration showing LD transfer (Reproduced with permission from Burnier *et al*. (69); used with permission of Mayo Foundation for Medical Education and Research. All rights reserved).

It was first described by Gerber *et al.* in 1988 for treating irreparable tears involving the posterosuperior rotator cuffs (supraspinatus and infraspinatus) with no axillary nerve palsy. The LD is detached from the anterior humeral shaft through a posteroinferior approach to the axillary region or endoscopically and transferred to the greater tuberosity under the deltoid. Thus, the LD is converted from an adductor and internal rotator to a humeral head depressor and external rotator (74), as shown in Fig. 3. The LD provides sufficient strength and amplitude with a good line of pull. It also has a consistent and mobile neurovascular pedicle. It is located in proximity to, but at a safe distance from, the terminal branches of the posterior cord of the brachial plexus (69, 70). The site of attachment of the LD tendon is up for debate. To maximize the tenodesis effect and complete coverage of the humeral head, a more anterior attachment to the front of the greater tuberosity or the subscapularis is indicated (75, 76). On the other hand, for better external rotation and elevation, a fixation into the infraspinatus insertion site is indicated (77).

Gerber *et al.* reported outcomes after LD tendon transfer using the open two-incision approach, finding that 74% of patients had good or excellent results at a mean follow-up of 10 years. The mean subjective shoulder value (SSV) significantly improved from 29 to 70%, along with increases in FF, ER, adduction, and strength (8). El-Azar *et al.* reported similar positive outcomes in 93 patients after 9 years, with improvements in pain relief, shoulder range of motion (ROM), and strength, a 10% failure rate, and 4% requiring conversion to reverse shoulder arthroplasty (78). Arthroscopic-assisted LD transfer has gained popularity for reducing deltoid damage, avoiding acromial osteotomy, shortening hospital stays, and improving cosmesis (79). Grimberg *et al.* reported that in 55 shoulders treated with arthroscopic-assisted LD transfer for irreparable posterosuperior RCT, SSV increased from 26 to 71%, the Constant score from 37 to 65.4%, and the activity score from 6.4 to 13.8% after 29 months, comparable to Gerber’s findings, with improvements in FF, ER, abduction, and strength (80). Castricini’s most extensive series study of 86 patients with a 3-year follow-up also showed that 82% achieved good or excellent outcomes (81).

Although this surgery is typically recommended for younger, active patients, arthroscopic-assisted LD transfer has shown favorable results in patients up to 69 years old, particularly those with higher preoperative Constant and Murley Scores (CMS) (76). This highlights that functional status before surgery is essential when determining surgical candidates. Other factors to consider are subscapularis tendon integrity and teres minor fatty infiltration.

### Lower trapezius tendon (LTT) transfer

LTT transfer was first described by Elhassan *et al.* in 2009 in a case report. This surgery was indicated in patients with paralytic shoulders who lacked external rotation instead of using LD tendon transfer (82). The LTT provides a more anatomic selection than LD as a tendon transfer for posterosuperior irreparable RCT, as it has a nearly identical line of pull as the infraspinatus (82).

Anatomically, the trapezius is divided into three parts: the superior, middle, and lower parts, which all act to elevate, retract, and externally rotate the scapula. The lower portions originate from T4-T12 spinous processes and insert into the medial parts of the acromion and spine of the scapula.

**Figure 4:**
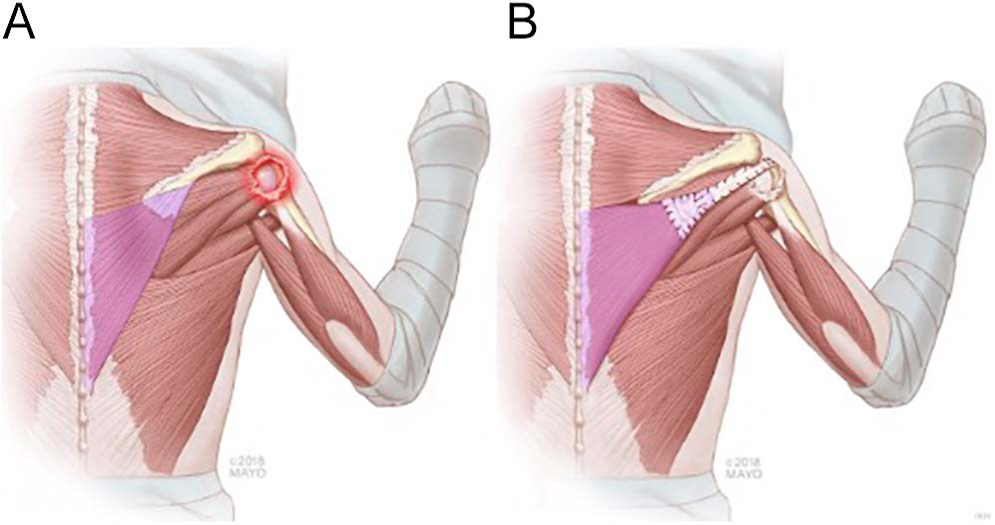
Illustration showing LT transfer (Reproduced with permission from Burnier *et al*. (69); used with permission of Mayo Foundation for Medical Education and Research. All rights reserved).

However, the LTT transfer is an indirect technique that requires the elongation of the tendon by a graft to reach the greater tuberosity. Initially, the open approach consists of a 5 cm vertical incision 1 cm medial to the medial scapular line; the tendon is detached from the spine of the scapula and dissected superiorly and medially. Deep dissection is usually avoided to prevent any iatrogenic damage to the spinal accessory nerve, which lies 2 cm medial to the medial border of the scapula underneath the trapezius tendon within the fascial layer. A limited exposure to the RC is done, and an Achilles tendon allograft is attached to the LTT by using two nonabsorbable sutures placed on each side of the LTT in a Krakow configuration. Afterward, the allograft is passed deep to the deltoid through the infraspinatus fascia and attached laterally to the posterosuperior part of the greater tuberosity using transosseous nonabsorbable sutures (Fig. 4) (83).

In 2016, Elhassan *et al.* reported findings of 33 patients followed up for 47 months following LT transfer. Their results showed that 32 patients had significant improvement in SSV (54 vs 78%; *P* < 0.01) DASH score (52 ± 19 vs 18 ± 10; *P* < 0.01), external rotation (50° range, 20°–70°; *P* < 0.01) with reported active muscle contraction of the transferred LT during shoulder external rotation. To note, patients with more than 60° forward flexion preoperatively improved ROM more than patients with less than 60° (84). Similar results were seen in patients undergoing arthroscopically assisted LT transfer with Achilles tendon allograft (85). Valenti *et al.* reported the outcomes of 14 patients following LT transfer with semitendinosus tendon. The Constant score increased from 35 ± 15 to 60 ± 9 points, and the mean SSV rose from 30 to 60% postoperatively compared to preoperatively (86). The findings from these studies suggest that LT transfer is an effective surgical intervention for enhancing shoulder function in patients with MRCT.

## SCR vs tendon transfer

Based on a systematic review of 20 studies, including 1,112 shoulders and a prospective cohort study with a mean follow-up of 6.3 years, SCR demonstrated better functional, patient-reported outcomes, and greater acromiohumeral distance compared to LD transfers in patients with massive irreparable posterosuperior RCT. In the systematic review, SCR showed significantly better scores in CMS (75.5 vs 65.6), ASES (83.4 vs 67.7), SSV (79.4 vs 64.4), and VAS (1.4 vs 2.8) compared to LD transfers. The prospective study also reported better outcomes for SCR patients in ASES (94.3 vs 72.1) and QuickDASH scores (8.8 vs 24.3). Both studies found no significant difference in terms of failure and reoperation rates (87, 88). Kadow *et al.* showed that there is a superiority in SCR when compared to LD transfer in terms of increased forward flexion preoperatively to postoperatively (52.4° vs 14.1). In comparison, LD transfer has a better improvement in the external rotation when compared to SCR (19.4° vs 0.8°) (89).

SCR is often more appropriate than tendon transfers for managing massive, irreparable RCTs in specific clinical scenarios. SCR is particularly indicated for patients with pseudoparalysis, as it has shown superior outcomes in restoring shoulder function compared to tendon transfers (90). It is also beneficial in cases where the primary concern is restoring glenohumeral stability and reversing proximal humeral migration, as it acts as a static stabilizer (91).

Biomechanical studies have demonstrated greater improvement in external rotation with LTT transfer than with LD transfer, leading to increased interest in using LTT for treating MRCT (82). A cadaveric study of eight fresh, frozen shoulders found that SCR and LTT significantly decreased superior translation compared to injured shoulders at 0° and 20° of abduction. LTT was superior in glenohumeral translation at all abduction angles. In addition, while both procedures reduced subacromial contact pressure, SCR provided a better subacromial contact area than LTT (92). Further evidence comes from a retrospective study of 58 patients with a mean follow-up of 39.3 months, which found that arthroscopically assisted LTT achieved significantly better outcomes in active shoulder range of motion, including forward elevation (165.7° vs 145.3°) and external rotation (51.7° vs 41.1°), ASES scores (84.8 vs 76.8), and patient satisfaction (8.9 vs 6.4) compared to arthroscopically assisted SCR (93). Moreover, the mean standardized cost of LTT transfer, including a 60-day workup and 90-day postoperative recovery, is $16,915 compared to $20,837 for SCR (82). This cost difference further underscores the potential benefits of LTT transfer over SCR for treating massive irreparable RCTs.

## Conclusion

Our review provides an overview of the current literature regarding SCR and TT, exploring their indications, surgical techniques, and outcomes. Both interventions present viable options for the management of massive irreparable RCTs; however, each has its benefits and drawbacks. Therefore, management criteria should be based on patient characteristics and clinical judgment while taking into consideration what each technique has to offer.

## ICMJE Statement of Interest

The authors declare that there is no conflict of interest that could be perceived as prejudicing the impartiality of the work reported.

## Funding Statement

The authors declare that no funds, grants, or other support were received during the preparation of this manuscript.
